# Stakeholders’ perception of a total market approach to HIV self-testing (HIVST) for the private sector in Nigeria

**DOI:** 10.1186/s12889-023-15352-0

**Published:** 2023-03-23

**Authors:** Dennis Aizobu, Omokhudu Idogho, Jennifer Anyanti, Godpower Omoregie, Boluwatife Adesina, Morgan Kabeer, Samuel Oyegunle, Serah Malaba, Akudo Ikpeazu, Yusuf H. Wada

**Affiliations:** 1 Society for Family Health, Abuja, Nigeria; 2 Busara Center for Behavioral Economics, Abuja, Nigeria; 3 Population Services International, Nairobi, Kenya; 4 National AIDS/STIs Control Programme, Abuja, Nigeria

**Keywords:** HIV, HIVST, TMA, Nigeria, Private sector, Market research

## Abstract

**Background:**

The continuous supply of affordable and quality HIV self-test (HIVST) is a key pillar toward achieving the global HIV 95–95-95 target in Nigeria. This was a descriptive qualitative study that explored private sector stakeholders’ perceptions of the enablers and barriers of the HIVST market in Nigeria.

**Methods:**

A total of 29 In-depth interviews (IDIs) were conducted with HIVST supply chain stakeholders and private sector providers (PPMVs and Community Pharmacies). Responses were analyzed using Nvivo software and we systematically developed a total market approach analysis for supply chain stakeholders and archetypes for community Pharmacies and PPMVs based on insights gathered from their journey map.

**Results:**

Challenges to the supply side dynamics include forecasting, point of care service delivery, the availability of free and subsidized HIVST kits in the market, neglect of private sector providers (Community Pharmacists and PPMVs) in the healthcare delivery system, limited demand for HIVST, and regulatory bottlenecks influences the overall market dynamics. High cost of the HIVST kit, which triggers low availability, accessibility and affordability from the demand side, depicts the need to understand the market dynamics. Addressing the barriers and optimizing the enablers of the three-model pharmacist and PPMV’s will change the market dynamic and service delivery to generate demand.

**Conclusion:**

To address challenges which already exist, the government need to revise the process guidelines for introducing new HIVST products in the Nigerian market, developing contingency plans to ensure the supply of HIVST remains sufficient when experiencing economic shocks, and create a sustainable roadmap toward optimizing the market for HIVST kits.

## Background

HIV is a high-burden disease in many countries of Sub-Saharan Africa. HIV testing is the first cascade of HIV care which is critical towards achieving epidemic control and attaining this ambitious global target. In many couples and sex partners, one or both partners do not know their HIV status [1]. The continuous supply of affordable and quality assured HIV self-test (HIVST) kits is a key pillar toward achieving the global UNAIDS HIV 95–95-95 targets and ensuring that over 95% of the people living with HIV (PLWHIV) know their status [2]. HIVST bypasses some barriers faced by conventional testing such as privacy concerns, stigma and inconvenience (e.g., time, transportation and locations) which hinder many from getting tested [3]. Provision of HIVST kits to address these barriers is important to reach other populations which are undiagnosed and accelerate progress in increasing the testing rate to reach those who have not yet been tested.

In Nigeria, HIV prevalence is 1.4% in the general adult population with an estimated 1.9 million persons living with HIV, with 71% (96 million) of the general adult population unaware of their HIV status [4]. Nigeria has included HIVST as part of its HIV testing strategies and has started to distribute HIVST kits through several distribution modalities [5]. However, the current distribution strategies for HIVST are either through primary or secondary distribution where it is given directly to the person who will perform the test or given to a relay person who will distribute it to one or many of his contacts such as key hidden populations, respectively [6–8]. Traditionally, these key populations are hard to reach through the primary distribution and may experience challenges such as violence from clients or partners through the secondary distribution strategy. Substantial evidence has demonstrated that HIVST through a properly designed distribution model increases consumer demand for HIV testing, especially among populations at risk for HIV, who are resistant to testing through conventional routes [9]. Further, several studies have shown that HIVST has high acceptability, increased testing frequency and promotes autonomy and discretion when distributed properly [10–14].

HIV testing products have been majorly government and public health facilities driven which have sidelined the private sector in Nigeria [15]. The global coronavirus (COVID-19) pandemic has shown the importance and capabilities of the private sector to augment government capacity within the public health system in developing and implementing self-care strategies and enhancing uptake in the country [16]. This calls for innovative and locally driven supply and demand strategies from the private sector to drive the HIVST market which provides opportunity for more access, affordability and availability.

This research aims to characterize the conditions and variables that influence the HIVST market according to the perceptions of a group of stakeholders who have extensive knowledge on total market approach. The study also aims to identify gaps in the processes and co-create new opportunities to develop actions to advance HIVST security in the private sector. Thus, we performed a market research activity for HIVST in Nigeria through a total market approach (TMA) lens which provides evidence-based data in driving access, availability and affordability of high-quality HIVST at the appropriate price points, with a focus on private sector driven market. All of these will also help create a more sustainable market for HIVST and protection for those at greatest risk for HIV and provide lessons for other countries with similar healthcare systems.

## Method

### Design

This was descriptive qualitative research exploring HIV self-testing (HIVST) market and opportunities for the private sector using a total market approach. It explores the enablers and barriers along the supply chain for importing, distributing, and selling HIVST kits in Nigeria, according to distributors, manufacturers, and HIVST managers. We further outline journey mapping for taking up and effectively using HIVST in the private sector among pharmacies/pharmacists and PPMVs.

### Recruitment of study participants

We conducted a total of 29 IDIs in this study and official communications were sent to already identified stakeholders which were purposely identified clearly explaining the research protocols and objectives. The inclusion criteria used in the study includes: staff aged 18 years or older, English speaking and willing to provide informed consent for the phone call interview. 9 IDIs were conducted with distributors, manufacturers, and HIVST managers across the country (HIVST managers were from public services who work for the ministries of health, national AIDS programs and regulatory agencies including the Pharmacy Council of Nigeria (PCN), National Agency for Food & Drug Administration (NAFDAC) and Medical Laboratory Science Council of Nigeria (MLSCN); 2 IDIs with distributors of healthcare products nationwide, 3 IDIs with manufacturers of quality-assured HIVST kits, 2IDIs with HIVST managers. We also performed 20 IDIs in three states with both Pharmacies and PPMVs in Urban and peri-urban areas (4 IDIs with pharmacies and 2 IDIs with PPMVs in Lagos, 6 IDIs with pharmacies and 1 IDI with PPMVs in Anambra, 4 IDIs with pharmacies and 3 IDIs with PPMVs in Kano state). All respondents were reassured that data that could potentially identify a person or location was anonymized in such a way that it could not be traced back to them. Electronic data were password protected.

### Data collection

Data was collected through a recorded phone call in English. During IDIs, respondents were categorically asked open-ended questions in pre-identified areas for discussion and analysis regarding HIVST in Nigeria. Community Pharmacists and PPMVs were asked to describe their experiences with selling HIVST kits and counseling customers who buy HIVST kits, while distributors and manufacturers were posed questions to uncover enablers and barriers along the supply chain. HIVST managers were asked about their experiences, opinions and perceptions regarding HIVST market development. All groups provided input on the policy recommendations for HIVST in the country and what challenges they foresee in distributing and selling HIVST in the private sector.

### Data management and analysis

All in-depth interviews (IDIs) were audio-recorded and we entered key information interview transcript into a spreadsheet. Thematic analysis (TA) was used to analyse and code the data using a qualitative software package – Nvivo. Framework analysis was employed through phases of familiarization, identifying a thematic framework, indexing charting and mapping and interpretation [17, 18].

Using information from the IDIs, we identified enablers and barriers to i) selling HIVST kits, ii) importing and distributing HIVST kits and we systematically reported them using a total market approach. For distributors, manufacturers, and HIVST managers, we compiled their insights and recommendations around key areas of interest. For service delivery outcomes relating information, we developed archetypes for Community Pharmacies and PPMVs based on insights from their journey mapping exercise. We further categories pharmacist who worked for community pharmacies into distinct groups based on their IDIs and other stakeholder’s perceptions which gives a broader insight into key drivers of their business. We also identified and synthesized the Nigeria’s HIVST market current use, unmet need, sources, total market approach indicators such as market size, market equity, sustainability and provide potential opportunities for the private sector towards expanding equitable and sustainable access to HIVST in Nigeria.

#### Ethical consideration

This study was approved by the two ethical review committee, the National Health Research Ethics Committee of Nigeria (NHREC) – NHREC/01/01/2007–29/06/2021 and the Nigerian Institute of Medical Research (NIMR) – IRB/21/043. Informed consent was obtained from participants after the benefits and objectives of the study had been fully read by them and all clarifications provided. All research activities were conducted in accordance with the Declarations of Helsinki and other policies and regulations required by the ethical committees. All respondents consented to participate and were free to voluntarily withdraw at any time with no attraction of penalty at any time of the research.

## Results

Overview on the current HIVST market and consumer demand in Nigeria:

Table 1 shows the HIVST products with either WHO Prequalification (PQ), expert review panel for diagnosis (ERPD), approval from founding member of International Medical Device Regulators Forum (IMDRF) or National Agency for Food & Drug Administration (NAFDAC) which are available in the Nigerian market. There are currently eight surveyed HIVST brand found in the Nigerian market with either of this approval, although there are also several unvalidated brands in the market.

**Table 1:** HIVST product kits found in Nigeria

**Test (Manufacturer)**	**Specimen**	**Approval**
INSTI HIV Self-Test (bioLytical Lab, Canada)	Blood	WHO PQ
OraQuick In-Home HIV Test (OraSure Technologies, USA)	Oral fluid	WHO PQ/
CHECKNOW HIV self-test (Abbott Rapid Diagostic JenaGmbH)	Blood	WHO PQ
Mylan HIV self-test (Atomo Diagnostics, Australia)	Blood	WHO PQ
Amethyst HIV 1& 2 Test Kit (Bedford Biotech Nigeria)	Saliva	NAFDAC
Health-Check 3 h-viral test (Artrion Lab Inc, Canada)	Blood	NAFDAC
Dr Greg’s HIV/AIDS home test kit (Winsag Intl LTD, Lagos)	Blood	NAFDAC
Voyage Rapid HIV home test kit (Nantong Voyage Medicals, China)	Blood	NAFDAC

### Market size dynamics

HIVST kits in the private sector make up a very small percentage of the overall market share. However, stakeholders see tremendous potential for HIVST kits in Nigeria. According to Ministry of Health officials:

*“When you have a country of about 200 million people, and you can get half of that population, if you can get 50% of the population using the HIV self-testing kits… If you get people to use it every three months, it would be a very good word for the private sector or for the business people promoting or selling the kits.”*

Although stakeholders are optimistic about the potential for HIVST kits in Nigeria, results from early roll-outs of selling HIVST kits in the private sector have been somewhat disappointing. A distributor says:

*“We recently began distributing oral-fluid-based HIVST kits in 13 states across Nigeria through retail and wholesale outlets and provided promotional materials and different merchandising products. After closely monitoring sales and operations, our results have not been as strong as they predicted, largely due to disruptions caused by terrorism and insecurity throughout the country. Despite these setbacks, we plan to roll out oral-fluid test kits to all 36 states.”*

To increase market growth, stakeholders reported that they need more engagement from state and local actors. A manufacturer says:

*“Community opinion leaders are better positioned to disseminate information on HIVST kits in their respective localities than members of the national government. Close collaboration with officials at lower tiers of government could help spread awareness, spark demand, and stimulate growth of HIVST kits in the private sector.”*

### Change in market equity: change in market shape in free, subsidized and unsubsidized HIVST products and services on the overall market

The majority of HIVST kits were reportedly purchased from distributors and manufacturers by the Ministry of Health, with funding from PEPFAR, the Global Fund, and Unitaid. Most of these HIVST kits are then distributed free of charge at health facilities and communities. Some pharmacies and private clinics also purchase HIVST kits, but these purchases are significantly smaller than public sector purchases, according to manufacturers.

Regarding end-users, stakeholders target HIVST kits to specific demographic groups. They target adolescents and men, as these groups do not frequently visit health facilities, and would likely find self-testing more appealing than conventional HIV testing. Stakeholders also target people living in *“crisis areas”* who cannot easily access healthcare services: A government official says:

*“…We target those who have a high-risk population or high prevalence. Those in parts of Benue and those in Borno. Some other parts, like parts of the Cross River, Akwa Ibom that have some people staying, living probably on the other side of the river. They have difficulties accessing our routine healthcare facilities. So, probably those are our first priority targets for HIVST kits.”*

Most stakeholders think reducing the price of HIVST kits could help stimulate demand, but they were against directly subsidizing the price at the Pharmacy or PPMV. During our interviews, manufacturers discussed related challenges in the past, where social marketing groups temporarily subsidized HIVST kits at pharmacies. A manufacturer says:

*“When the subsidy was removed, consumers thought the manufacturers and distributors increased the price. Moreover, while the subsidy was in place, pharmacies would not purchase HIVST kits from distributors since they could get a cheaper price from these marketing groups, impacting the distributor’s profits and private sector demand creation initiatives.”*

Majority of our stakeholders think subsidies of this nature are unsustainable and therefore subsidies could be used for promotion and advertising, making kits more affordable for distributors and consumers offered with vouchers or coupons for purchasing HIVST kits at pharmacies and PPMVs, and these coupons can be paid for by subsidies: A distributor says:

*“Providing people with the voucher system where they can get so much off may be a better way [to subsidize] because you don’t entrust the price of the product in the pharmacy, but you give people the coupon to get it at a half price or something like that….”*

### Change in market accessibility

**Fig. 1:**
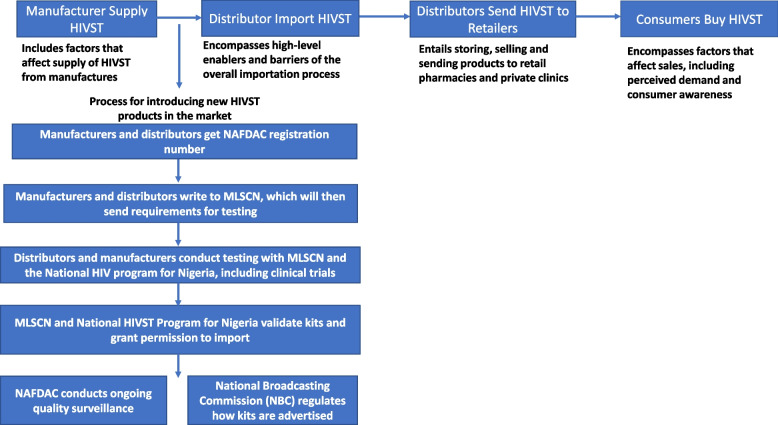
Market accessibility archetype showing steps involved in importing, distributing, and selling HIVST kits in Nigeria (including the process for introducing new HIVST products in the market)

Figure 1 show the market accessibility archetype across the supply chain to the customers who buy the HIVST kit in Nigeria. Stakeholders reported to see tremendous potential for introducing new HIVST in Nigeria and were asked to describe how they perceived an ideal process for it. Majority of them describe the process in similar ways which was used to design an archetype (Fig. 1) with the private sector and regulatory agencies playing distinct roles across the supply chain to meet the needs of HIVST for the private sector to thrive. They described the process from how the manufacturers and distributors can be able to navigate the supply chain which they narrated will increase investment, improve standards and ensure stronger checks on the whole HIVST market.

### Market sustainability

**Fig. 2:**
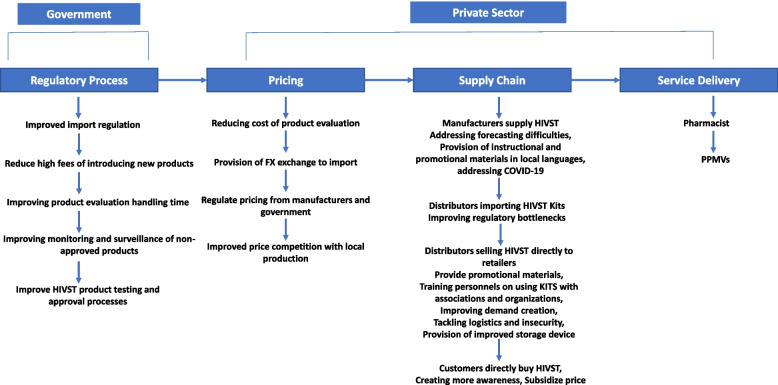
Stakeholders archetype of improved/sustainable HIVST market in Nigeria

The Fig. 2 shows that most HIVST are being driven using a private sector distribution model (supply chain, pricing and service delivery) with influence from government (regulatory process) providing regulation which enable provision of more high-quality HIVST kits.. The private sector distribution sustainability is an ongoing discussion for stakeholders in Nigeria. However, most stakeholders think reducing the price of HIVST kits could help stimulate demand, but they were against directly subsidizing the price at the pharmacy or PPMV. During our interviews, manufacturers discussed related challenges in the past, where social marketing or donor driven groups temporarily subsidized HIVST kits at pharmacies. Nevertheless, unvalidated HIVST kits are spreading throughout Nigeria because the charges associated with introducing new medical products are too high. It is expensive to register a new HIVST product and conduct clinical testing in accordance with National Agency for Food and Drug Administration (NAFDAC) and Medical Laboratory Science Council of Nigeria (MLSCN) regulations. As a result, distributors and retailers are bringing low-quality HIVST kits into the market without going through the registration process.

### HIVST supply chain in Nigeria

**Fig. 3:**
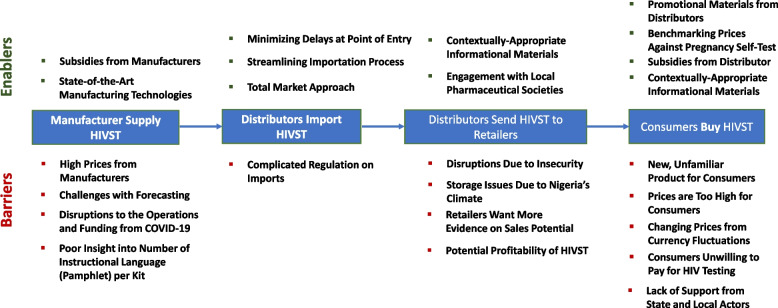
Enablers and Barriers of Supply Chain of HIVST in Nigeria

In our study, we interviewed various stakeholders to distill the supply chain for HIVST kits in Nigeria. We also identify various factors which may have pose barriers at each step in the supply chain, impacting HIVST kit supply and distribution in Nigeria, and thus preventing consumers from accessing kits. Conversely, certain factors may enable the supply and distribution of HIVST kits. From our interviews, we identified these enablers and barriers along the supply chain, which are shown in Fig. 3 and we divided our distilled result into four distinct:

Further, we asked manufacturers, distributors, and HIVST managers to describe the process for introducing a new HIVST product in the market, then explain the most difficult step in the process along the supply chain. Most participants described issues with the testing process, specifically: manufacturers, distributors, and HIVST managers all agree that testing is too complicated and expensive. According to a manufacturer:

*“The process is not only complicated and time consuming, it is also expensive because before the product is made available say in a country like Kenya or Nigeria, there is a fee of up to $100,000 for testing, $25,000 factory inspection fee, and daily fees of $500-$2,000 for officials who make visits to the factory. This deters manufactures from registering their products, and even makes the products more expensive and harder to maintain stores for manufacturers.”*

HIVST kits fail testing because they cannot withstand high temperatures in Nigeria. According to a HIVST manager:

*“One challenge I see is if they are not in country manufacturers. There are kits that fail evaluation if they don’t understand our weather or the climate in this part of the world. Probably, you do something in Geneva without putting the very high temperature in Africa in mind, and you bring it into this part of the world, it might end up failing evaluation. If manufactured in country, then that challenge might not necessarily apply.”*

### Potentials for the Nigerian HIVST Market through the private sector

**Fig. 4:**
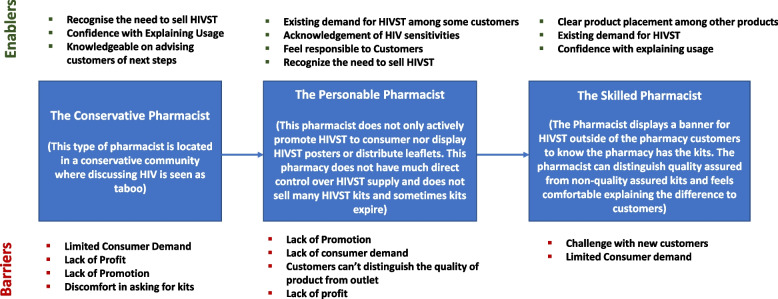
Enablers and barriers for new model pharmacist for HIVST in Nigeria
  B HIVST ARCHETYPES FOR PPMVs:
    The PPMVs we interviewed explained that there is existing awareness and demand among their customers.
    We developed archetypes (Fig. 5) which serves as potentials to unlock the HIVST in Nigeria. Majority of the PPMVs interview think it is not their responsibility to build awareness and demand for HIVST kits. They do not hang any posters or distribute fliers, nor do they actively recommend HIVST kits to their customers.

In this study, we developed a journey map which shows potentials (challenges and opportunities) for HIVST kits in the private sector at pharmacies and patent and proprietary medicine vendors (PPMVs) stores They include:

I Service delivery:
  In our study, we interviewed stakeholders to develop HIVST archetypes for the pharmacist and PPMVs to describe the behavioral characteristics for key groups and also highlights the potentials (enablers and barriers) along the step to selling HIVST in Nigeria.
  A HIVST ARCHETYPES FOR PHARMACISTS
    We interviewed Pharmacist as service providers of HIVST in the private sector. Figure 4 shows the archetypes for pharmacists and describe the enablers and barriers of the behavioral characteristics for key groups in selling HIVST. They were three groups of pharmacists; the Conservative Pharmacist, Personable Pharmacist and Skilled Pharmacist based on how they offer service delivery of HIVST to consumers.

**Fig. 5:**
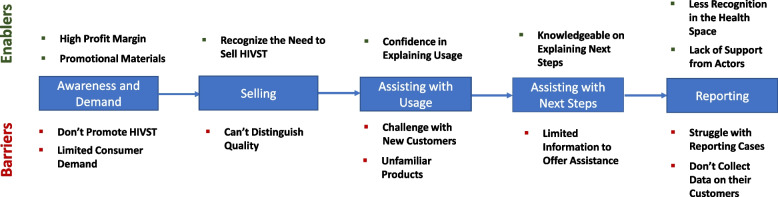
Enablers and barriers among PPMVs to selling HIVST in Nigeria
    As one PPMV stated:
*“It's not my responsibility to create awareness. My responsibility is to stock and sell.”*
    Further, many PPMVs think customers demand HIVST kits because they are self-aware and health-conscious. Some customers know the factors that put them at risk of HIV, and they understand that their behavior may put them at risk. In an effort to stay healthy, they want to know their HIV status, and therefore, they demand HIVST kits. Conversely, a few PPMVs interviewed think that their customers are aware, but they doubt the accuracy of HIVST kits, which may stem demand. As one PPMV explained:
*“…. the only sales challenges we have is that of the new users doubting the effectiveness of the self-test kits, and we have to assure and reassure them of the effectiveness.”*
    Among customers who are willing to engage with the PPMV, some do not understand the instructions for using HIVST kits, even after PPMVs demonstrations and explain correct usage. PPMVs attribute this limited understanding to language barriers and low education levels. They also said that some of their customers do not have much experience with self-testing products. Some must spend extra time with these customers, repeating instructions and carefully answering questions.
    According to one PPMV:
    “…some clients, because of their educational background… you need to explain in the layman's language for them to understand.”
II Supply chain
  A Manufacturers Supply HIVST Kits
    In our interviews, the manufacturers we spoke explain the *“state-of-the-art”* processes and technologies they have and how they are capable of meeting demand for HIVST kits in Nigeria, as may be required. However, these manufacturing capabilities can be expensive, and distributors said prices from manufacturers are too high, which impacts the final retail price of HIVST kits.
    1. Forecasting difficulties: although manufacturers possess the capabilities to meet demand, many countries like Nigeria still face challenges with delays and stock outs, which manufacturers attribute to difficulties with forecasting. As one manufacturer explained:
*“Forecasting [is the biggest barrier in the supply chain] …the ability of countries to be able to predict accurately when they are going to need the product and how much they are going to need… in order to ensure timely order fulfillment, it should be important that we know in advance as much as possible how many tests countries want and when they want them”*
    2. Instructional materials and packaging: From our interviews, instructional materials and packaging manufactured are majorly manufactured in English. The majority of the manufacturers we interviewed agree that production of HIVST instructional materials in our local languages will generate demand.
    3. COVID-19 Pandemic: From our interviews, the majority of the manufacturers say that the COVID-19 pandemic has impacted on the supply and demand of HIVST kits. Fewer distributors are purchasing HIVST kits from manufacturers. As one manufacturer stated:
*“Sadly, in the last 18 months or something, I’ve seen a downturn in demand for self-tests. I think it’s got something to do with…the impact of COVID-19 on the infrastructure. I think it’s got something to do with funding.”*
  B Distributors Import HIVST Kits
    Our respondents (manufacturers) reported that the Federal Ministry of Health is taking a “total market approach (TMA)” to HIVST kit supply and distribution, whereby they let the private sector manage importation and distribution within certain regulations, with the government playing a key role in the overall coordination.
    As one Ministry of Health official explained:
*“We are also looking at the TMA approach in-country. The private sector is probably interested in the HIV sector, selling kits in the country. The sector would get regulations, do some evaluation, and have the kits in the country. The private sector would distribute kits to any state while the government just coordinates...”*
    However, most stakeholders think regulations on imports are too complicated. As a result, there are few HIVST products in the market, which has effectively created an oligopoly with little price competition. According to one Ministry of Health official:
*“...knowing the different agencies regulating the importation has to be made simple so that more testing kits can come into the country. That can help lower the prices where we have limited quantities, you understand? …. if we can get the market to be flooded with good commodities, that is one way that we can use to address pricing.”*
    In response, regulators and Ministry of Health officials are actively working to improve the importation process. The Ministry of Health recognizes that different agencies have similar mandates around managing and regulating imports, and they are currently bringing these agencies together to identify overlap, remove needless complications, and “*streamline”* processes. Regulators also try to avoid causing delays at ports of entry by minimizing processing times:
*“....we try to support [by ensuring] facilitation of this product is done very quickly, so that when goods arrive, we don’t want issues of goods being tied down at the port when there are delays….”*
  C Distributors Send HIVST Kits to Retailers:
    I Promotional materials: In our interviews, manufacturers provide contextually-appropriate informational and promotional materials to help stimulate demand for HIVST kits among pharmacies and PPMVs. One manufacturer explained.
*“…my organization has partnered with the Ministry of Health in some countries to run radio advertisements and raise awareness and we also distribute posters and other print materials through partnerships with local pharmaceutical societies to put some branding into the pharmacy to support the product.”*
    II Trainings: Retailers and distributors reported that manufacturers do organize trainings for them through their partnerships with pharmaceutical societies. Despite these efforts, many stakeholders think that demand for HIVST kits is low among pharmacists and PPMVs. They explained how HIVST kits are relatively new to the market in Nigeria, and retailers are not sure if HIVST kits will be profitable for their businesses. According to one distributor:
*“... because it's a new product, remember that we're dealing with people in the private sector, they're kind of wary. Nobody wants to be the guinea pig. In any product, usually, they like to see that other people are selling and making profit before they start stocking it.”*
    III Stocking HIVST: Pharmacists and PPMVs are *“business people,”* and they will only stock and sell HIVST kits if they are profitable. As one Ministry of Health official stated:
      “Will [HIVST kits] be profitable to [pharmacists and PPMVs]? If the answer is yes, they’ll go for it, but if the answer is no, it might be challenging.”
    IV Insecurity and violence: Stakeholders reported that they face several challenges with distribution due to insecurity and violence. They reported that Insecurity and violence throughout Nigeria impacts transportation of pharmaceutical such as HIVST. According to one distributor:
*“So we haven't seen that rate of acceleration…. because of different reasons. One of them is security issues… we've had to slow down the reach of some products in certain areas. In the southeast, we experience the issues of the separatist people. We had issues with terrorists, you know, attacks in the north.”*
    V Storage: Majority of the stakeholders reported that storage temperature is a key factor for distribution. The hot and humid climate in Nigeria also makes it difficult for distributors and retailers to store HIVST kits for prolonged periods of time. As one regulator observed:
      “We also have issues of keeping these kits in proper temperature that they do not deteriorate.”
  D Customers Buy HIVST Kits:
    I Awareness: Majority of the stakeholders we spoke to are all involved in spreading awareness of HIVST kits among consumers through a number of different channels. Manufacturers and distributors provide retailers with posters and banners to display in their stores, often translated into local languages. As one distributor described:
*“... product banners [are] usually hung in front of the retail outlets, so that when consumers come, they get to see the information on the product. Manufacturers and Ministry of Health officials also advertise HIVST kits on the radio”*
    II Support: Stakeholders also receive little support from state and local actors in their efforts to spread awareness. One regulator explained:
*“…it is important to improve engagement with community opinion leaders at the state level [and] local government [level], who can assist with disseminating information on HIVST kits throughout their localities.”*
    III Pricing: Stakeholders also agree that the price of HIVST kits is too high for most consumers, even though some distributors said they subsidize the final price. These high prices impact demand for HIVST kits, especially among young students. Currently, fluctuations exacerbate this problem, giving consumers the impression that prices are continuously changing:
      “…$1 USD is ₦500 NGN, and that can change overnight to ₦350 NGN. As distributors, we are forced to change the price, which affects the end users.”
III Regulatory Processes:
  In our interviews, majority of stakeholders think unregulated kits are spreading through the Nigerian market because:
  1. Import regulation: Majority of the stakeholders reported that the import regulation is too complicated, so there are few kits available on the market, creating an oligopoly with little price competition. A HIVST manager:
*“I think the risk is seen is knowing the different agencies regulating the importation has to be made simple so that more testing kits can come into the country. That can help lower the prices. Where we have limited quantities, you understand, the pricing will remain high... if we can get the market to be flooded with good commodities, that is one way that we can use to address pricing.”*
  2. High fees: Majority reported that fees associated with introducing new medical products are too high; it is expensive to register a new product and conduct clinical testing in accordance with NAFDAC and MLSCN regulations. As a result, distributors and retailers are bringing kits into the market without going through the registration process. A distributor says:
*“They are affecting business because if one doesn’t have enough money to pay for registration and purchase products legitimately, they will be forced to buy the products from the black market where no one can verify the quality or source...”*
  2. Evaluation processes:
    A HISVST manager highlights that:
    “For distributors, it boils down to the evaluation process, which I think the government is handling at this time. We had a meeting with them. That was the first major challenge they raised, that the cost of evaluation was too high, which I think has been brought down now.”
  3. Robust monitoring and surveillance:
    The majority of our respondents reported that robust monitoring and surveillance of the HIVST market will help reduce the number of unregistered HIVST kits on the market. A HIVST manager says:
    “So, one is to try and bring down the prices of the approved ones and open up the market and also monitor that open market so that there are no leakages in our process that allow people to be able to access things in the open market.”
  4. CGMP HIVST product testing: A manufacturer says:
    “I would tell you that COVID-19 has really brought a lot of attention to laboratory services in Nigeria when it comes to the actual testing and marketing of laboratory test kits. I think the government is really focusing on it now, and we as regulators have set up our game as well in terms of validation and ruling out of sub-standard kits in the country because your kits, their test results are as good as the kit that you market…. I think especially marketers in Nigeria have started to up their game, and we are now seeing better compliance to regulatory requirements.”
IV Pricing: Our respondents (stakeholders) reported that there are a number of factors affecting the pricing of HIVST:
  1. Distributors claim that prices from manufacturers are too high, despite manufacturers subsidizing the price for kits sold across Africa from their own profits. According to a distributor:
    “Because we get it at a very high price from the manufacturer, we had to do a lot of promotion where we subsidize the price with our number-one selling kits countrywide. This is to encourage the uptake by customers and actually to support that drive and to push the product in the market and as our best-selling product in Nigeria.”
  2. The cost of evaluation for distributors and manufacturers is too high. A HIVST manager says:
    “For distributors, it boils down to the evaluation process, which I think the government is handling at this time. We had a meeting with them, that was the first major challenge they raised. That the cost of evaluation was too high, which I think has been brought down now.”
  3. Pharmacists/PPMV owners are business people at the end of the day, so prices need to be high enough to earn a profit. A HIVST manager says:
    “A lot of them [pharmacists and PPMV owners] are business people. They will look at the cost. Will it be profitable to them? If the answer is, yes they’ll go for it but if the answer is no, it might be challenging.”
  4. Regulation is too complicated, so there are few kits available on the market, creating an oligopoly with little price competition. A HIVST manager says that there is need for regulation procedures that need to be made simple for prices to be lower.
    “I think the risk is seen is knowing the different agencies regulating the importation has to be made simple so that more testing kits can come into the country. That can help lower the prices. Where we have limited quantities, the pricing will remain high... if we can get the market flooded with good commodities that is one way that we can use to address pricing.”
  5. Uniform pricing: Majority of the manufacturers says there are plans to have an in-country price uniformity for HIVST to thrive. A HIVST manager says:
    “...we started a meeting on that with the stakeholders in the country. We were looking at if we can have a uniform price, between one thousand Naira and one five. I think there were some challenges because of the evaluation processes that had been done over the years in pricing.”

## Discussion

Our study reported for the first time, a comprehensive overview of the HIVST market dynamics at a national level. This is coupled with a deep understanding of the demand side through the enablers and barriers of the key players to recognize the evolving market actors and actions, aspirations, ability to pay and consumer behaviors. The study result provides new evidence to access bottlenecks and key drivers, which we believe will provide an effective evidence-based data for decision makers and stakeholders understand and prioritize regulatory and policy actions that will support market development.

Our study has revealed that factors hampering the market growth from the supply side dynamics includes forecasting, point of care service delivery, dominance of free and socially marketed HIVST on the market, neglected private sector in the public health system, limited demand for HIVST among key populations and the general population at large, and regulatory bottlenecks influencing the overall market dynamics. This finding, supported by previous research through the Self-Testing Africa (STAR) initiative project [19, 20], highlights the critical challenges faced in the HIVST sustainable market implementation, positive policy environment and social challenges limiting scale-up and acceleration of market development work.

Other challenges or barriers faced across the supply chain for HIVST in this study stemmed from low demand for point of care delivery from the manufacturers such as difficulty in reading instructional materials (e.g., leaflets and inserts), promotional materials (banners, roll-up items and stickers), and storage conditions (kits requiring special temperature and capacity development for HIVST. This is similar to challenges faced in African countries with implementing rapid diagnostic tests such as malaria in the private sector [21–23]. Therefore, there is a need to better support the needed paradigm shift, an upward policy development that should accommodate local language development for instructional and promotional items, logistical and capacity development to support for demand creation that will promote local buy-in and can inform behavioral change communication strategies targeting providers.

In our study, stakeholders complained about subsidy and donor related challenges in the past, where social marketing groups temporarily subsidized HIVST kits at pharmacies. These issues can be addressed through models such as the condom social marketing approach where government, donors and private sector work effectively to ensure a steady supply along with a sustainable market [24]. In addition to the issues around the supply side dynamics, HIVST is still relatively new in Nigeria with complex regulatory processes [25]. This has brought about many inexpensive, substandard, low-quality HIVST kits on the market in Nigeria that have not been verified by the government. Previous studies have demonstrated that substandard health products have been a topical issue in Nigeria and do pose a serious health concern to patient’s safety and health outcomes and public trust in the healthcare system [26–29]. Failing to resolve supply chain bottlenecks may increase demand for what stakeholders refer to as *“black market”* HIVST kits. These kits are referred to as the black market because they are sold illegally without regulatory approval for use in the country. However, addressing bottlenecks perceived by the stakeholders have particular relevance in cases such as the black market HIVST kits to thrive. This offers new evidence for policy makers and regulators to monitor and react to changes that might affect quality, availability, affordability and creating dialogues among stakeholders to allow more targeted investments in HIVST. Various stakeholders in our study reported difference in service delivery provided by Pharmacies and PPMVs which have impact in the market for HIVST. The study reported problems with limited consumer knowledge of HIVST and presumptive or incorrect treatment provision among service providers. This is similar to studies on promoting malaria RTDs using the private sector and have been addressed using interventions such as health promotion that include behavior change and educational activities targeted to consumers and providers to promote confidence in testing and linkage services to provide actions to both a positive and negative test result, be it to administer treatment or to refer to another health provider [30, 31]. Despite this, stakeholders also reported the need for continuous capacity building for service providers to distinguish quality, data collection to feed national HIV data, provision of evidence-based information and next steps in linking care. Health seeking behavior of many people have changed towards self-care in the country due to growing self-awareness on the internet such as e-pharmacy, but at the policy level this is yet to change significantly. Technology can be used to influence regulatory processes, manufacturers, clinical and other stakeholders practice and help shape the overall market for HIVST. Looking at other countries for example, technology have be leveraged to connect service providers for capacity building and HIVST users to counseling services. The Challenge Fund has explored using unstructured supplementary service data (USSD) codes to help pharmacists in Kenya with reporting around HIVST kit distribution and vending machines such as in workplaces [32]. This concept can also be adapted to provide HIVST users with information to improve privacy. Online ordering could be particularly effective at reaching residents of urban and peri-urban areas, where internet and electricity are relatively strong and stable, compared to rural areas. Therefore, more advocacies, research and policy work would be needed to bring knowledge of health markets and health systems to policy dialogue and strengthen and facilitate how private sectors providers such as can be part of the national continuum of HIV care management for epidemic control. There are limitations in our study that need to be taken into consideration when interpreting the findings. First, respondents from the service delivery side (pharmacies and PPMVs) were drawn from three states; Anambra, Kano and Lagos States using a convenient sampling. This method was more cost effective and time efficient, but might have resulted in the biases of our sample. Further, these pharmacists and PPMV owners may have a level of awareness around HIV and HIVST that isn’t necessarily representative of the average for the population. We also have relatively few PPMV owners compared to pharmacists. We were also unable to recruit many pharmacists and PPMV owners in peri-urban areas who sold HIVST; all IDIs in Kano state were conducted with pharmacists and PPMV owners in urban areas. Similarly, interviewed stakeholders such as distributors, manufacturers, regulators, and Ministry of Health officials might have likely exhibited biases that they may or may not be aware in presenting some of their views as absolutely correct, such as their views on enablers and barriers to HIVST take up and use among end-users, while they were only giving their opinions. We attempted to overcome all these limitations by organizing a co-creation exercise aimed at identifying key stakeholders in the development and delivery of HIVST. They were further selected based on their expertise and can provide valuable insights into the research question.

## Conclusion

Our study highlights key challenges and important opportunities to improve the supply and sustainability of HIVST market in Nigeria. The low availability and accessibility of HIVST may be attributed to lack of demand, supply side issues and costs. The role of government in regulatory oversight and improving the quality, availability, affordability and accessibility in the private sector should be strengthened. There is also need for more strengthened market factors for HIVST such as affordable pricing, regulations and additional approved kits for use in the private sector which will increase choice and competition for a healthier HIV self-test market in Nigeria. New strategic ways such as government revising the process guidelines for introducing new HIVST products on the Nigerian market, developing contingency plans to ensure the supply of HIVST remains sufficient when experiencing economic shocks, subsidize prices of HIVST at the manufacturer level with the support of funders towards creating a more buoyant private sector. In addition, coupons and vouchers present another potentially viable option for subsidizing HIVST which include endorsements from doctors or nurses to help build consumer confidence and product demand for HIVST.

## Data Availability

The dataset generated and analyzed during the current study are available on reasonable request from the corresponding author.
